# Loss of binding antibodies against rabies in a vaccinated dog population in Flores Island, Indonesia

**DOI:** 10.1371/journal.pntd.0009688

**Published:** 2021-09-07

**Authors:** Ewaldus Wera, Charlotte Warembourg, Petrus M. Bulu, Maria M. Siko, Salome Dürr

**Affiliations:** 1 Animal Health Study Program, Kupang State Agricultural Polytechnic (Politeknik Pertanian Negeri Kupang), Kupang, Indonesia; 2 Veterinary Public Health Institute, Vetsuisse Faculty, University of Bern, Bern, Switzerland; 3 Animal Health Division, Agricultural Department of Sikka Regency, Maumere, Indonesia; Universidad Nacional Mayor de San Marcos, PERU

## Abstract

Effective parenteral vaccines are available to control rabies in dogs. While such vaccines are successfully used worldwide, the period between vaccine boosters required to guarantee protection of the population against rabies varies between vaccines and populations. In Flores Island, Indonesia, internationally and locally produced rabies vaccines are used during annual vaccination campaigns of predominantly free-roaming owned domestic dogs. The study objective was to identify the duration of the presence and factors associated with the loss of adequate level of binding antibodies (≥0.5 EU/ml) following rabies vaccination in a domestic dog population on Flores Island. A total of 171 dogs that developed an antibody titre higher or equal to 0.5 EU/ml 30 days after vaccination (D30), were repeatedly sampled at day 90, 180, 270, and 360 after vaccination. On the day of vaccination (D0), an interview was performed with dog owners to collect information on dog characteristics (age, sex, body condition score (BCS)), history of rabies vaccination, kind of daily food, frequency of feeding, and origin of the dog. Serum samples were collected and the level of antibodies was quantitatively assessed using ELISA tests. Dogs were categorized as having an adequate level of binding antibodies (≥0.5 EU/ml) or inadequate level of binding antibodies (<0.5 EU/ml) at each time points examined. A total of 115, 72, 23, and 31 dogs were sampled at D90, D180, D270, and D360, respectively, with the highest proportion of antibodies ≥ 0.5 EU/ml (58%, 95% CI, 49–67%) at D90, which reduced gradually until D360 (35%, 95% CI, 19–52%). Multivariable logistic regression models showed that loss of adequate level of binding antibodies is significantly associated with dogs having no history of vaccination or vaccination applied more than 12 months before D0, being less than 12 months of age, and having a poor BCS. These results highlight the importance of BCS regarding the immune response duration and provide insights into frequency of vaccination campaigns required for the internationally available vaccine used on Flores Island. For dogs without vaccination history or vaccination being applied more than 12 months before D0, a booster is recommended within 3 months (a largest drop of antibodies was detected within the first 90 days) after the first vaccination to guarantee measurable protection of the population that lasts at least for one year.

## Author summary

Rabies is a zoonotic disease, caused by a lyssavirus, resulting in thousands of deaths every year, particularly in low-resource endemic regions in Asia and Africa. High quality rabies vaccines have been available since decades to guarantee protection of the dog population against rabies. Furthermore, maintaining a high vaccination coverage and herd immunity until the subsequent vaccination campaign is essential for preventing circulation of rabies virus within dog populations. However, the percentage of dogs that maintain an adequate level of rabies antibodies one year after vaccination and factors influencing such a maintenance is unknown for the dog populations on Flores Island, Indonesia. This study aimed at understanding the risk factors associated with loss of adequate level of binding antibodies amongst vaccinated dogs that developed antibody titre ≥0.5 EU/ml after parenteral vaccination, following a longitudinal study over one year. The study identified body condition score of the dogs, their previous vaccination, and their age as factors that influence the maintenance of an adequate level of antibodies. For dogs without previous vaccination and vaccination being applied more than 12 months ago, a booster is recommended within 3 months after the first vaccination to guarantee an antibody levels that lasts for at least one year. In addition, a low BCS should be considered as one of the risk factors of losing measurable immunity. Therefore, improving the dogs’ poor BCS should also be promoted to enhance immune response and ensure longer presence of antibodies against rabies.

## Introduction

Rabies is one of the oldest zoonotic diseases and has a case fatality rate of almost 100% both in animals and humans [1,2]. People become infected through close contact with saliva of infected animals, mostly via bites [1]. More than 100,000 humans exposed to rabid animals are reported annually worldwide, resulting in approximately 60,000 deaths [3]. Over 95% of these cases were associated with rabid dogs [3]. Prevention of rabies in humans can be achieved by post exposure prophylaxis, including wound treatment and vaccination after bite by a suspected rabid animal [1]. However, the most effective and sustainable way is to eliminate the disease by mass vaccination within the reservoir population, notably the domestic dog populations [4].

Vaccination against rabies in dogs is efficient and available since decades. It has been demonstrated in various settings that rabies can be eliminated from a reservoir population by mass vaccination. One of the first successful stories of a mass vaccination program in a dog population occurred in the city of Memphis (Shelby County) in the United States in 1948, in which the number of human cases reduced to zero within five months after a mass vaccination campaign in dogs [5]. Other success stories of mass vaccination in dog population were reported from Latin American countries [6], Africa [7,8], and Asia [9]. In Asia, for example, mass vaccination of dogs successfully decreased the human rabies incidence in Bali Island, Indonesia [9], Bhutan [10], and Philippines [11]. These success stories depend not only on well-organized dog mass vaccination programmes and high vaccination coverage, but also on the effectiveness of vaccines to protect dogs against rabies in natural setting [12].

Flores Island, Indonesia, has been endemic of canine rabies since 1998, and reports 15 human deaths annually [13]. With the objective of controlling rabies, annual dog vaccination campaigns are publicly funded in which Rabisin vaccine (Boehringer Ingelheim) have been used in the last couple of years. Maintaining a high vaccination coverage and herd immunity until the subsequent vaccination campaign is essential for preventing circulation of rabies virus within dog populations. However, the duration of the maintenance of immunity in the Flores Island dog populations have not been studied yet.

Although it is known that cellular immunity plays a role for the protection against rabies after vaccination [14], the immune status of an individual can be only quantified in a practical way by measuring the circulating antibodies. Rabies sero-prevalence studies have previously been conducted in Asia and Europe [15,16,17,18]. These studies focused on determining the pattern of immunogenicity amongst different dog age groups, however they lacked investigation of other risk factors associated with loss of adequate level of antibodies against rabies after vaccination. Several studies examined factors influencing the development of immunity against rabies after vaccination in dogs, and found that age at the time of vaccination, timing of blood sampling, and breed of dogs were associated with the level of antibodies against rabies [19–23]. However, none of these studies were dedicated to explore these aspects in free-roaming domestic dogs (FRDD).

FRDD can be defined as being ownerless or owned dogs that are unconfined at least part of the time. On Flores Island, Indonesia–as in most rabies endemic countries–dogs are predominantly kept as owned FRDD [24]. The owners typically feed their dogs with leftovers, such as rice, for their daily food, which generally is of low protein value and not adequate for canine nutrition [25]. As a consequence, most of the dogs have a poor body condition score (BCS). A poor body condition score (BCS) is expected to negatively influencing the immune response of dogs after vaccination, as it could be shown that the lymphocyte counts in dogs with lower BCS are less than those dogs with ideal BCS [12].

The current study aimed at understanding the risk factors associated with loss of adequate level of binding antibodies, taken as a measurable metric for their immunity, amongst vaccinated dogs that developed immunity after parenteral vaccination on Flores Island, following a longitudinal study approach. The results of this study will inform policy makers to better plan frequencies of vaccination campaigns and improve their effectiveness.

## Materials and methods

### Ethics statement

Approval to conduct the study was obtained from the Animal Ethics Commission of the Veterinary Medicine Faculty, Nusa Cendana University (Protocol KEH/FKH/NPEH/2019/009). Informed consent for participation was obtained from dog owners before conducting the study.

### Study area

**Fig 1:**
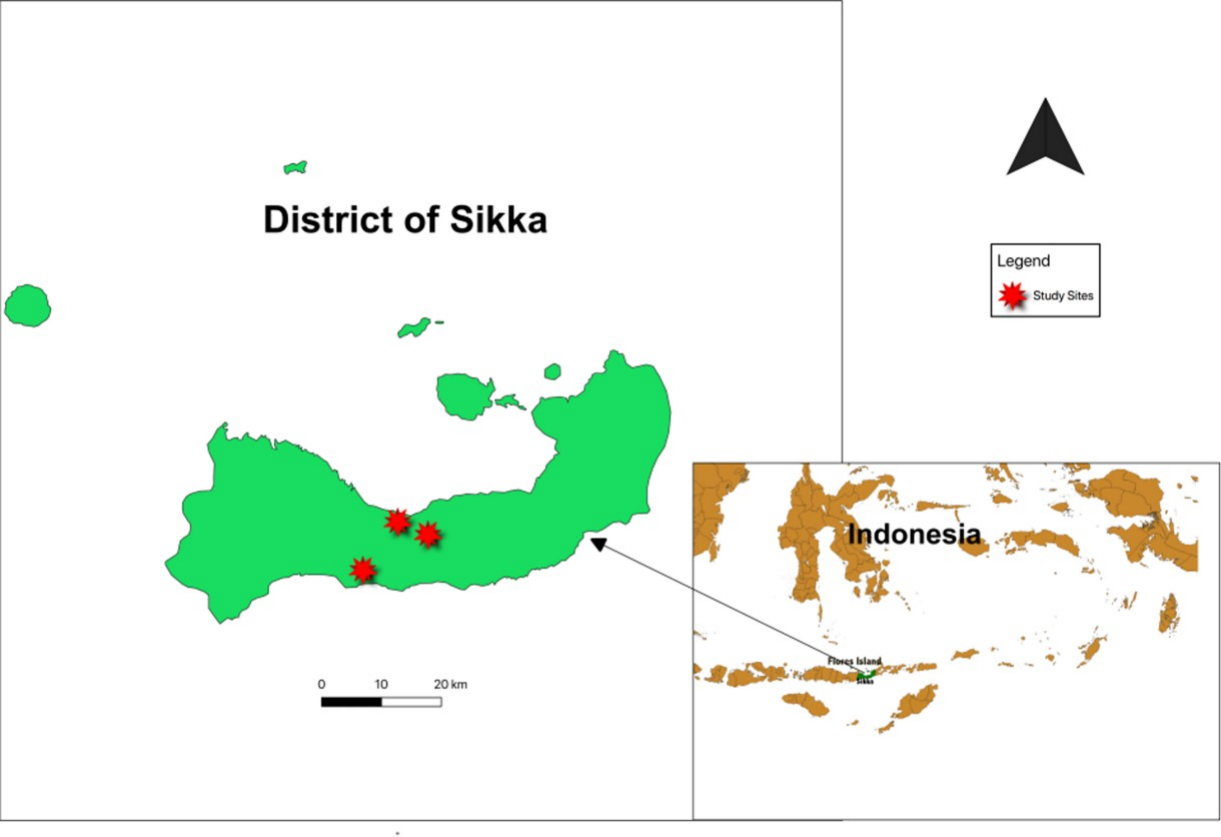
Study area. Base layer: https://www.statsilk.com/maps/download–free–shapefile–maps.

Blood sampling and questionnaire distribution were conducted in households with dogs in rural (Pogon and Hepang) and urban (Habi) areas in Sikka Regency, Flores Island, Indonesia between July 2018 and August 2019 (Fig 1). The regency was selected following an initial survey conducted by the principal author, which indicated the high prevalence of human rabies on Island of Flores [24]. Sikka is located in the eastern part of Flores Island and covers an area of 15,624 km^2^. The regency is divided into 351 villages, with a human population of more than 300,000 inhabitants (census data of 2010) and an owned dog population of greater than 37,000 [24]. Many of the villages in the regency are categorized as rural areas and are only accessible by foot or with high-clearance vehicles or motor bikes. As in other regencies in Flores Island, agriculture is the most important socio-economic activity (production of coconut, corn, groundnut, cocoa, coffee, potato, and paddy), in which dogs are used to guard the crops. Most dogs are owned and roam freely day and night [26]. Dogs have a high cultural and economic value in Flores Island, as they provide a source of animal protein in addition to their guarding capacities. Dog meat is a popular menu item in certain traditional ceremonies of the island.

### Sampling design

Since this study was part of a larger dog ecology study, the sampling design was driven by the necessity to study the complete dog network in a delimited area [27,28]. Therefore, we defined an area of 1 km^2^, both in rural and urban sites, where we aimed at including all dogs. During the blood sampling, sick and/or pregnant dogs were excluded from the study to avoid miscarriage due to stress.

### Data collection

#### Blood samples

At day 0 (D0), dog blood samples were taken from 256 dogs and each dog was vaccinated against rabies immediately after the blood sampling. The dogs were vaccinated intramuscularly with 0.5 ml of Rabisin vaccine (Boehringer Ingelheim). Of 256 sampled dogs at D0, 187 (73.1%) dogs could be followed-up 30 days afterwards (D30), of which 171 (91.4%) dogs had developed virus binding antibody levels of ≥0.5 Equivalent Unit per ml serum (EU/ml). As the aim of the study was to identify risk factors for losing immunity defined as an adequate binding antibody titre ≥0.5EU/ml at different time point amongst vaccination, the analysis was conducted prospectively on the 171 dogs that developed an antibody titre ≥0.5 EU/ml at D30. Blood was further sampled from the exact same dogs at 90 (D90), 180 (D180), 270 (D270), and 360 (D360) days after vaccination, whenever the dog was present for sampling. Blood (3–5 ml) was sampled from *v*. *cephalica* and put into a blood tube. The blood tubes were transported to the animal health laboratory of Agricultural Department of Sikka Regency. At the same day of sampling, the full blood was separated by centrifugation to extract the serum, which was then dispensed into 3 ml labelled Eppendorf tubes. The tubes were stored at +4°C until shipping to veterinary laboratory (Disease Investigation Center, Denpasar) in Bali, where ELISA (Enzyme linked immunosorbent assays) tests were performed to determine the rabies antibody titres.

### Dog characteristics

Information on dog characteristics, history of vaccination, kind of daily food, frequency of feeding and BCS was collected during the interviews at D0. The BCS ranged from 1–5, which was later on categorized as poor for values 1–2 and good for values 3–5 for the data analysis. During the following visits, the number of study dogs culled, sold, pregnant, sick, unable to handle, or absent from home during the visit–and therefore not sampled at that time point–was recorded. The interview were conducted in Bahasa by the researcher team.

### Laboratory tests

Serum samples were tested for the presence of rabies antibodies using a Rabies ELISA kit produced by Pusvetma, Surabaya, Indonesia (http://pusvetma.ditjenpkh.pertanian.go.id/main.php?page=detail_produk&id=28). This ELISA kit was validated against ELISA Platelia II Rabies kit (Bio-Rad, France) for the detection of rabies binding antibodies, with sensitivity, specificity, and Kappa coefficient being 96.8%, 73.5%, and 0.68, respectively [29]. Samples were tested following the manufacturer’s instructions. In brief, serum samples were inactivated at 56°C for 30 minutes to inactivate potential pathogens that contaminated samples and may harm laboratory technicians. Each sample was diluted by mixing of a 2.5 μl serum and 247.5 μl PBS-Tween and 100 μl per sample were distributed in one well each of microplates coated with whole rabies virus (Pasteur Virus strain). The microplate was sealed and incubated at 37°C for 60 minutes and then washed four times with PBS-Tween. Afterwards, conjugate protein A labelled peroxidase was added into wells of microplate (100 μl/well) and sealed. The microplate was incubated at 37°C for 60 minutes and then washed four times with PBS-Tween. Substrate solution was added 100 μl per well. The microplate was placed in dark room for approximately 10 minutes, before stopper solution was added. Optical density was measured at 405 nm with an ELISA reader. Samples were categorized as negative, i.e. having inadequate level of binding antibodies, for ELISA if the titre was below 0.5 EU/ml.

### Statistical analysis

Descriptive statistics were performed to describe the dog population characteristics for each blood sampling time point (D30, D90, D180, D270 and D360). The proportion of dogs having lost adequate level of binding antibodies at each time point was calculated by dividing the number of dogs with a negative ELISA test by the respective number of sampled dogs. Chi-square test was used to compare the proportion of dogs losing adequate level of rabies binding antibodies in regards to the dog individual characteristics. The association between investigated factors (Table 1, independent variables) and loss of adequate level of binding antibodies after vaccination (outcome variable) was assessed using univariable logistic regression analyses for each time point separately. Four multivariable logistic regression analyses (one each per time point) were subsequently conducted to determine the influence of each independent variable to the outcome after adjusting for other variables. All independent variables that had p-values lower than or equal to 0.25 in the univariable analyses were subsequently included in the initial models for the multivariable analyses [30]. Prior to the multivariable analyses, multicollinearity between the independent variables (all of categorical nature) selected from the univariable analyses were checked using Chi-Square tests. For correlated variables (Chi-Square Test p-value <0.05), the variable which showed the higher association with the outcome variable was considered in the multivariable analyses while the other one was excluded. The final multivariable logistic models were derived by backward stepwise elimination in which variables with a p-value > 0.05 were excluded one-by-one in each step. The Hosmer-Lemeshow goodness-of-fit test was performed to determine the fit of the final models to the data [30]. The final models were considered a good fit for the data if the p-value of the Hosmer-Lemeshow test was greater than 0.05. SPSS version 19 was used for data analysis.

**Table 1:** Demographic characteristics of dog surveyed in Flores Island, Indonesia at day 30 after vaccination (n = 171).

	Frequency (n)	Percentage (%)
Sex:		
Female	118	69.0
Male	53	31.0
Age:		
<12 months	100	58.5
> = 12 months	71	41.5
Breed:		
Local breed	170	99.4
Other	1	0.6
Residential area:		
Urban	67	39.2
Rural	104	60.8
History of rabies vaccination before D0^a^:		
<12 months	55	32.2
Never or >12 months	116	67.8
Origin of dogs:		
Born in house	83	48.5
Given or bought	88	51.5
Kind of daily food:		
Leftovers	159	93.0
Other^b^	12	7.0
Frequency of food:		
< 3 times per day	77	45.0
> = 3 times per day	94	55.0
Body condition score ^c^:		
Poor	95	55.6
Good	76	44.4

^a^ D0 is the day of vaccination within this study.

^b^Other daily food like rice, corn, fish.

^c^BCS was range 1–5 which was categorized as poor if score less than 3 and good if score 3–5.

## Results

### Dog characteristics

The demographic characteristics of the 171 dogs having built an adequate level of binding antibodies at the start of the study (D30) is provided in Table 1. The majority of the dogs was female (69.0%), aged less than 12 months (58.5%), and had either no previous vaccination or was vaccinated more than 12 months before D0 (67.8%) according to the owners statements. Most dogs were local breed (99.4%), fed with leftovers (93.0%), and had a poor BCS of less than 3 (55.6%). These characteristics were comparable for each blood sampling time point, except for sampling D270 where more males were sampled than females (Tables 2 and 3).

**Table 2:** Frequency (n) and percentage (n/N) of dogs losing adequate level binding antibodies 90 (D90) and 180 (D180) days after vaccination, stratified by different demographic characteristics of the dogs. The influence of demographic parameters were explored by univariable logistic regression analyses.

Variables	Day 90 (N = 115)						Day 180 (N = 72)					
	Frequency (N)	Loss of adequate level of binding antibodies^f^ (n)	Percentage (%)	OR	95% CI	P-value*	Frequency (N)	Loss of adequate level of binding antibodies^f^ (n)	Percentage (%)	OR	95% CI	P-value
Sex:						0.454						0.770
Male	34	16	47.1	1.36	0.61–3.05		20	10	50.0	0.85	0.30–2.40	
Female	81	32	39.5	1.00			52	28	53.8	1.00		
Age^a^:						**0.037**						**0.011**
<12 months	66	33	50.0	2.27	1.04–4.92		37	25	67.6	3.53	1.33–9.32	
> = 12 months	49	15	30.6	1.00			35	13	37.1	1.00		
Breed:						na						na
Local breed	115	48	41.7				72	38	52.8			
Other	0	0	0				0	0	0			
Residential area^a^:						**0.217**						0.565
Urban	46	16	34.8	1.00			23	11	47.8	1.00		
Rural	69	32	45.1	1.62	0.75–3.50		49	27	55.1	1.34	0.49–3.61	
History of rabies vaccination^b^ before D0^c^:						**0.026**						**0.022**
<12 months	40	11	27.5	1.00			30	8	33.3	1.00		
Never / > = 12 months	75	37	49.3	2.57	1.12–5.88		42	30	62.5	3.33	1.19–9.34	
Origin of dogs:						0.605^g^						**0.177**
Born in house	54	24	44.4	1.00			30	13	43.3	1.00		
Given or bought	38	16	42.1	0.90	0.39–2.10		42	25	59.5	1.92	0.74–4.97	
Kind of daily food:						**0.078** ^g^						0.378
Leftovers	106	47	44.3	6.37	0.77–52.77		68	35	51.5	0.35	0.04–3.57	
Other^d^	9	1	11.1	1.00			4	3	75.0	1.00		
Frequency of food:						0.618 ^g^						0.487
<3 times per day	45	18	40.0	1.00			37	21	56.8	1.00		
> = 3 times per day	70	30	42.9	2.67	0.23–30.80		35	17	48.6	0.72	0.28–1.82	
Body condition score^e^:						**0.025**						**0.040**
Poor	62	32	51.6	2.40	1.11–5.19		47	29	61.7	2.86	1.05–7.84	
Good	52	16	30.8	1.00			25	9	36.0	1.00		

OR = Odds ratio; CI = Confidence interval; na = no statistic are computed because 100% of dogs are local breeds; p–value shown in bold represents p< = 0.25; these variables were used in the subsequent multivariable logistic regression analysis.

^a^Exclude from subsequent multivariable logistic regression analysis at D90 as the variable age and residential of dogs were detected to be significantly correlated with history of vaccination and BCS, respectively (p–value<0.05).

^b^Exclude from subsequent multivariable logistic regression analysis at D180 as the variable history of vaccination was detected to be significantly correlated with age (p–value<0.05).

^c^ D0 is the day of vaccination in this study

^d^Other daily food include rice, corn, or fish.

^e^BCS was range 1–5 which was categorized as poor if score less than 3 and good if score 3–5.

^f^Rabies binding antibody titre of < 0.5EU/ml.

^g^Fisher x^2^ square test

**Table 3:** Frequency (n) and percentage (n/N) of dogs losing adequate level binding antibodies 270 (D270) and 360 (D360) days after vaccination, stratified by different demographic characteristics of the dogs. The influence of demographic parameters were explored by univariable logistic regression analyses.

Variables	Day 270 (N = 23)						Day 360 (N = 31)					
	Frequency (N)	Loss of adequate level of binding antibodies^d^ (n)	Percentage (%)	OR	95% CI	P-value	Frequency (N)	Loss of adequate level of binding antibodies^d^ (n)	Percentage (%)	OR	95% CI	P-value
Sex:						0.531*						0.516*
Male	19	11	57.9	0.46	0.04–5.26		7	5	71.4	1.36	0.61–3.05	
Female	4	3	75.0	1.00			24	15	62.5	1.00		
Age:						0.017*						0.052*
<12 months	15	12	80.0	12.00	1.56–92.29		13	11	84.6	2.27	1.04–4.92	
> = 12 months	8	2	25.0	1.00			18	9	50.0	1.00		
Breed:						1.000*						0.645*
Local breed	22	13	59.1	Na			30	19	63.3			
Other	1	1	100				1	1	100.0			
Residential area:						0.400*						0.477*
Urban	12	6	50.0	1.00			14	8	57.1	1.62	0.75–3.50	
Rural	11	8	72.7	2.67	0.47–15.25		17	12	70.6	1.00		
History of rabies vaccination before D0^a^:						**0.136**						**0.037**
<12 months	4	1	25.0	1.00	1.14–5.49		7	2	28.6	2.50	1.14–5.49	
Never / > = 12 months	19	13	68.4	6.50	0.56–76.17		24	18	75.0	1.00		
Origin of dogs:						0.657*						0.532*
Born in house	16	9	56.3	1.00			13	12	66.7	1.00		
Given or bought	7	5	71.4	1.94	0.29–13.19		18	8	61.5	0.90	0.39–2.10	
Kind of daily food:						0.136*						0.254*
Leftovers	19	13	68.4	6.5	0.55–76.18		28	17	60.7	6.37	0.77–2.77	
Other^b^	4	1	25.0	1.00			3	3	100.0	1.00		
Frequency of food:						0.907*						0.553*
<3 times per day	15	9	62.5	1.11	0.19–6.49		16	10	66.7	1.00		
> = 3 times per day	8	5	60.0	1.00			15	10	62.5	2.67	0.23–3.80	
Body condition score^c^						0.029*						**0.050**
Poor	12	10	83.3	8.75	1.24–61.68		16	13	81.3	2.40	1.11–5.19	
Good	11	4	36.4	1.00			15	7	46.7	1.00		

OR = Odds ratio; CI = Confidence interval; na = no statistic are computed because 100% of dogs are local breeds

*Fisher x^2^ square test; p–value shown in bold represents p< = 0.25; these variables were used in the subsequent multivariable logistic regression analysis.

^a^D0 is the day of vaccination within this study

^b^Other daily food like rice, corn, or fish.

^c^BCS was range 1–5 which was categorized as poor if score less than 3 and good if score 3–5.

^d^Rabies binding antibody titre of < 0.5EU/ml.

### Loss of adequate level of binding antibodies after vaccination

**Fig 2:**
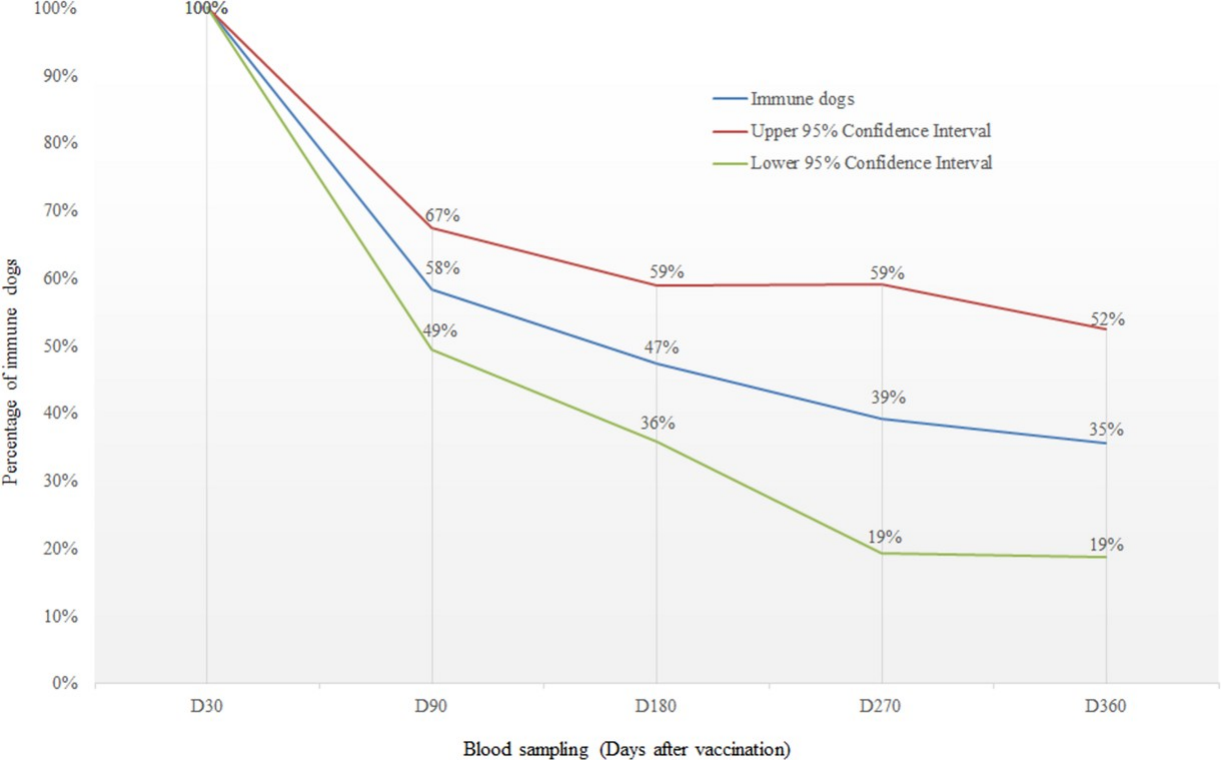
Percentage of dogs developed binding antibody titres ≥0.5EU/ml at days 90 (n = 115), 180 (n = 72), 270 (n = 23), and 360 (n = 32) after vaccination surveyed in Flores Island, Indonesia in 2018–2019. The dogs developed binding antibody titres ≥0.5EU/ml at day 30 (n = 171) were taken as study population.

A total of 115, 72, 23, and 31 dogs were sampled at D90, D180, D270, and D360, respectively. The highest proportion of dogs maintaining binding antibody titres ≥0.5EU/ml was observed at D90 (58%, 95% CI: 49–67%), and then reduced gradually until D360 (35%, 95% CI: 19–52%) after vaccination (Fig 2).

The loss of adequate level of binding antibodies after vaccination differed among dog characteristics according to the univariable analyses (Tables 2–4). At each time point, the proportion of dogs losing adequate level of binding antibodies was significantly higher in dogs younger than 12 months compared with dogs aged 12 months or more (p<0.05). Among dogs younger than 12 months, the proportion of dogs having lost adequate level of binding antibodies was found to be 50.0% (95% CI: 38–62%) at D90 and increased thereafter until the highest proportion at D360 (84.6%, 95% CI: 59–93%) (Tables 2 and 3). In comparison, for dogs older than 12 months, loss of adequate level of binding antibodies was observed in 30.6% (95% CI: 18–44%) of the dogs at D90 and 50.0% (95% CI: 27–73%) at D360. Furthermore, among dogs without history of vaccination or those having been vaccinated more than 12 months before D0, a higher proportion lost adequate level of binding antibodies compared to their counterparts, but the difference was only statistically significant at D90, D180, and D360. Amongst dogs without history of vaccination, the proportion of dogs losing adequate level of binding antibodies was found to be 49.3% (95% CI: 38–61%) at D90 and increased to the highest proportion at D360 (75.0%; 95% CI: 58–92%) (Tables 2 and 3), whereas for dogs having been vaccinated less than 12 months before D0 these proportions were found to be 27.5% (95% CI: 14–41%) and 28.6% (95% CI: 9–76%), respectively. Similarly, the BCS was detected as significant parameter influencing the loss of adequate level of binding antibodies after vaccination for each time point. The proportion of dogs having lost adequate level of binding antibodies in dogs with a BCS lower than 3 was found to be 51.6% (95% CI: 39–64%), 61.7% (95% CI: 48–76%), 83.3% (95% CI: 49–92%), and 81.3% (95% CI: 58–90%) at D90, D180, D270, and D360, respectively, compared to 30.8% (95% CI: 18–43%), 36.0% (95% CI: 17–55%), 36.4% (95% CI: 13–74%), and 46.7% (95% CI: 21–72%) in those with a BCS equal or higher than 3. The proportion of dogs losing adequate level of binding antibodies for dogs in rural areas was higher than dogs in urban areas at each blood sampling time point, but the differences were not statistically significant (Tables 2 and 3). The proportion of dogs losing adequate level of binding antibodies among dogs that were obtained as gifts (given by relative families) or bought from traditional markets tended to be larger at D180 and D270 and lower at D90 and D360 compared to their counterparts, but these differences were found to be statistically non-significant. The influence of breed could not be analysed due to the very low number of dogs being other than local breed.

**Table 4:** Determinants associated with loss of adequate level of binding antibodies 90, 180, 270 and 360 days after rabies vaccination in dogs on Flores Island, Indonesia, using multivariable logistic regression analysis. All dogs showed an adequate rabies binding antibody titre of ≥ 0.5EU/ml 30 days after vaccination.

Variables	Day 90 (N = 115)		Day 180 (N = 72)		Day 270 (N = 23)		Day 360 (N = 31)	
	OR (95% CI)	p-value	OR (95% CI)	p-value	OR (95% CI	p-value	OR (95% CI	p-value
Age:								
<12 months			3.63 (1.33–9.99)	0.012	14.51 (1.29–172.97)	0.034		
> = 12 months			1.00		1.00			
History of rabies vaccination before D0^a^:								
> = 12 months	2.39 (1.02–5.57)	0.044					2.3 8.69 (1.08–70.16)	0.043
Never / <12 months	1.00						1.00	
Body condition score (BCS)^b^:								
Poor	2.32 (1.06–5.09)^c^	0.036	2.98 (1.03–8.61)	0.044	10.69 (0.93–122.63)	0.057	5.68 (0.92–35.09)	0.062
Good	1.00		1.00		1.00		1.00	

OR = Odds ratio; CI = Confidence interval.

^a^D0 is the day of vaccination within this study

^b^BCS was range 1–5 which was categorized as poor if score less than 3 and good if score 3–5.

^c^Information on the BCS was missed for 1 dog

The Hosmer–Lemeshow goodness–of–fit test p–value for the model day 90, 180, 270, and 360 was 1.00, 0.99, 0.52, and 0.49, respectively

Significant association between independent variables were detected for age and history of vaccination (Chi-Square Test p-value = 0.006 and <0.001 for the D90 and D180 model, respectively), between residential area of dogs and history of vaccination (Chi-Square Test p-value < 0.001 for the D180 model), and between residential area of dogs and BCS (Chi-Square Test p-value < 0.001 for the D90 model). The variables of age and residential area of dogs at D90 and history of vaccination at D180 were thus excluded from subsequent multivariable analyses (Table 2). No significant association between independent variables were observed for the D270 and D360 model (Chi-Square Test p-value >0.05).

The multivariable logistic regression models showed that the proportion of dogs losing adequate level of binding antibodies was significantly and positively associated with dogs without history of vaccination or having been vaccinated more than 12 months before D0, dogs less than 12 months, and dogs with poor BCS (Table 4). The significance of these factors depends on the time point of blood samplings. For example, the results for D90 showed a significant association with the BCS and the history of vaccination before D0. Dogs with poor BCS were two times more likely (OR = 2.32; 95% CI = 1.06–5.09) to lose adequate level of binding antibodies compared to those dogs with good BCS. Similarly, dogs without history of vaccination or having been vaccinated more than 12 months before D0 were two times more likely (OR = 2.39; 95% CI = 1.02–5.57) to lose adequate level of binding antibodies compared to their counterparts. Age and history of vaccination before D0 had a significant contribution on the proportion of dog losing adequate level of binding antibodies at D270 and D360, respectively. BCS was the only variable that was associated with loss of adequate level of binding antibodies after rabies vaccination at each time point, although for D270 and D360 the p-value was slightly above the defined threshold of being statistically significant (Table 4).

### Loss of follow-up

Of the 171 immune dogs in the cohort (those developed binding antibody titres ≥0.5EU/ml at D30), 56 (32.7%), 99 (57.9%), 147 (85.9%), and 138 (80.7%) were excluded from the analysis at D90, D180, D270, and D360, respectively. The reasons for the lost follow-up include the absence of dog owners and/or dogs at home during the time of the visit, the dog could not be handled, pregnant, sick, death of the dog due to diseases and culling for meat source purpose.

## Discussion

In this longitudinal study, we investigated antibody levels after rabies vaccination in FRDD from 30 up to 360 days after vaccination under field conditions in both urban and rural areas in Flores Island Indonesia. We found that the proportion of dogs having an adequate level of rabies binding antibodies out of those that developed such a level 30 days after vaccination, dropped massively after 60 days (D90) to 58%, and then further at each time point of investigation to 35% at D360. The trend of reducing rabies antibodies after vaccination is well documented in the literature and those findings overlap with what was found in the present study [12,17,31–34]. A study conducted in Bali, Indonesia, found that the proportion of dogs having a titre higher than 0.5 IU/ml reduced from >90% to 60–80% at 30 and 360 days after vaccination, respectively [12]. Similarly, Minke et al [34] studied the antibody titre in a group of 30 laboratory dogs vaccinated with Rabisin rabies vaccine and found that the proportion of dogs with a titre ≥ 0.5 IU/ml declined sharply from 93% at 28 days to 40% at 120 days post vaccination. Furthermore, Suzuki et al [32] studied the immune response of 236 vaccinated domestic dogs under field condition in Bolivia and found that the proportion of dogs with a protective antibodies seven months after vaccination was only 58%.

The natural loss of rabies antibodies in dogs after vaccination [32] can be influenced by many factors, such as health status of vaccinated dogs [12], type of the vaccine used [34], and age of the dogs at the time of vaccination [21]. In the current study, we demonstrated that dogs aged less than one year at the time of vaccination were more likely to lose adequate level of binding antibodies at D180 and D270 compared to their counterparts. It is well documented in the literature that dogs less than one year of age have an increased risk of having a poor antibody response [19–21]. Two factors could contribute to this finding. First, the maternal antibodies titre received from vaccinated dams limits effective immune response in puppies [35,36], and it is common in Flores Island that dog owners vaccinate their reproductive female dogs [24]. Second, younger dogs have a lower chance to having already received one or several vaccine doses before the start of our study, depending on the frequency of rabies vaccination campaigns undertaken in the region. In Sikka Regency, vaccination campaigns are conducted annually, with the aim of vaccinating all dogs aged more than three months once per year. Dogs born during or after the annual vaccination campaign will be vaccinated the following year, which is the majority of those aged less than one year at the time of vaccination in our study. As a consequence, dogs aged less than one year received their first dose of rabies vaccine at D0, while their counterparts probably have already been vaccinated in the past. This is in line with our finding that the reported history of vaccination significantly influenced the loss of adequate level of binding antibodies after vaccination for some time points investigated (D90 and D360). The presence of T-cell memory that have been activated in the previous vaccination, contributes to a more rapid and profound immune response in dogs with previous vaccination [37]. As a consequence, higher levels of antibodies are produced after booster vaccination that then maintain for a longer duration at a level >0.5 EU/ml [19,21].

Our study highlights the importance of BCS on the immune response of dogs after vaccination. Dogs with low BCS were more likely to lose adequate level of binding antibodies than their counterpart. Early loss of adequate level of binding antibodies in dogs with low BCS score could be related with a number of factors such as parasitic burden, nutritional and general health status [12,38,39]. Parasitic infection and malnutrition is common in FRDD in Flores Island, as it is in other rabies endemic areas in developing countries, where dog owners may neither routinely apply anti-parasite treatments nor feed the dogs with adequate food [40]. Our study found that 93% of dog owners stated to daily feed their dogs with leftovers, such as rice, which generally is of low protein value compared to adequate nutrition for canines [25]. This finding suggests that the maintenance of adequate level of binding antibodies following rabies vaccination depends on the nutritional and health status of FRDD, even for vaccination with high quality vaccines (such as Rabisin).

We found that the majority (65%) of dogs that developed binding antibody titre ≥0.5 EU/ml at D30 failed to maintain this level until 360 days after rabies vaccination. This proportion is higher than the previous study carried out on Bali Island, Indonesia, where only 20–40% of vaccinated dogs were observed to lose neutralizing antibodies until D360 [12]. The difference between the two studies may have occurred due to different test systems used, which was an ELISA in the current study compared to the fluorescent antibody virus neutralisation (FAVN) test in the study in Bali [12]. The FAVN test is a serum neutralization test with higher resource demands than for the ELISA [41], however with higher sensitivity for antibody detection compared to ELISA, depending on the threshold used [42,43]. Another reason for the lower proportion of protected dogs at D360 compared to the study in Bali [12] might lay in the difference of the dog management and BCS in the study populations, with dogs in Bali most probably have a higher BCS than dogs kept in Flores due to higher average income of the owners.

Furthermore, the present study found that at D90, almost 50% of dogs without reported vaccination history has binding antibody levels <0.5 EU/ml (Table 2). The drop of the antibody level below the threshold of 0.5 EU/ml [44] does not automatically lead to a loss of immunity, as documented by Dodds et al [14] and Aubert [45]. Dodds et al [14] reported that 80% of vaccinated dogs without detectable antibodies were fully protected against a rabies-virus challenge. Aubert [45] demonstrated that animals developing rabies neutralizing antibodies above a threshold of 0.5 IU/ml after rabies vaccination, have a high probability of surviving after a contact with rabies virus, regardless of the level of neutralizing antibodies at the time of exposure. This phenomenon is due to the shift of humoral towards memory cellular immune response developed by T and B lymphocytes, which are responsible for a faster and more effective immune response in the event of rabies virus exposure [14]. We therefore underestimate the actual immunity of the dogs when focusing on the assessment of the humoral response only, which is however the method applicable in practice. Therefore, in order to guarantee a measurable immune response that last for at least one year, revaccination within three months after the first vaccination is highly recommended in dogs without any previous vaccination.

A limitation of the current study is the high number of dogs lost for the followed-up after D30, which lead to a wide confidence interval of immunity coverage over time (Fig 2). In addition, to have a complete understanding on the impact of different type vaccines on the immune response, it would be interesting to compare the humoral response obtained in dogs vaccinated with locally produced vaccines with those in dogs vaccinated with internationally produced vaccines in a future study.

## Conclusion

The results of this study provide knowledge on the loss of adequate level of rabies binding antibodies of FRDD and on risk factors associated with it, from 30 to 360 days after vaccination. The results highlight the importance of BCS, vaccination history, and age of dogs for the maintenance of an adequate level of rabies binding antibodies and provide insights into required frequency of rabies vaccination campaigns in FRDD on Flores Island. For dogs without vaccination history and vaccination being applied more than 12 months before D0, a booster is recommended within 3 months after the first vaccination to guarantee development of detectable antibodies lasting for at least one year. In addition, good dog management should be recommended to improve BCS of the animals, which would enhance maintenance of binding antibodies.
